# Supplementary material for: Genome-Wide Identification and Expression Analysis of the Aquaporin Gene Family in Lycium barbarum during Fruit Ripening and Seedling Response to Heat Stress
Source: Curr Issues Mol Biol. 2022 Nov 28;44(12):5933–48. doi: 10.3390/cimb44120404 (PMC9777030; doi:10.3390/cimb44120404)
Supplement: Supplementary file 1 [file cimb-44-00404-s001.zip › Table S1.pdf]

Genes	Primers（5'-3'）	TM value(℃)	Length（bp）
LbNIP5;1	F GGTGGCGTTACTGTTCCTTC	58.85	149
	R ACTGTGGCTCCAACTGCTAT	59.01	
LbNIP6;1	F AGTGGGCTGGCTGTAATGAT	59.08	109
	R TCCAGGGGAAATGCTTGAGA	58.63	
LbNIP4;3	F TGGGGAGCAGTTGTAATGGT	58.93	171
	R TAAAGTACCGCCTGCCAGAA	59.02	
LbNIP4;2	F CAGTGTGCCATCTTCAGCTC	58.91	101
	R TCACGACCTGCCTTTTCTCT	58.95	
LbNIP4;4	F GGGGTCGATTCTAGCAAGCT	59.53	151
	R AACTCCGCAAACAACGAACA	58.91	
LbNIP4;1	F TCCATACAGCCTCCGATTCC	58.95	129
	R TCCCTATGACCTCCGCAATC	58.95	
LbNIP3;1	F CAACGTCGTAACACTACCCG	58.67	420
	R CCTGTTAGTGGCCCAGAGAA	59.02	
LbNIP1;2	F AACTTGCTGGTCTTGCTGTG	58.97	115
	R CTTGACACTATTGCTGGGCC	58.90	
LbNIP2;1	F AAACACGATCACTTTGCCGG	59.41	122
	R ATTGGCCCGCTAAACATCAC	58.90	
LbTIP5;1	F CATGTGAATCCGGCTGTGAC	59.27	107
	R CAAGCCATGACAGACCCAAG	58.83	
LbTIP2;3	F TTGGAACCATTGCACCCATG	59.02	109
	R GCTGGCCCAAATGATCTAGC	59.04	
LbTIP2;1	F GGTTTCCATTGCCGCTAACA	59.11	109
	R AATCCAGTAGAAGAGGCCGG	58.88	
LbTIP2;4	F GGCTCCATTGTTGCTTGCTA	58.82	102
	R CACAACTCCTTCAGCAGCTC	58.84	
LbTIP2;2	F CTGCTGTCACCTTCGGATTG	58.92	111
	R ATTTGAGGAGGAGGCAAGCT	59.00	
LbTIP4;1	F GGAGCTTCAATGAACCCTGC	59.19	109
	R CAGCAAGACCACCACCAATC	59.12	
LbTIP1;1	F GTTTTCGCAGGTCAGGGTTC	59.41	142
	R CGGAGATGTTAGCACCAACG	59.00	
LbTIP3;1	F TGGGCTGGTCTACACTGTTT	58.86	147
	R CTTGCTGGATTCATGGACGC	59.62	
LbTIP3;2	F CATTGCCCCTCTTGCCATTG	59.82	148
	R GCCCAACCAGTAGATCCAGT	59.09	
LbXIP1;6	F AGCAGGCCTCCTTGTGTTTA	59.23	146

	R GCCCAACCCAAAAGATCCAA	58.65	
LbXIP1;2	F ACTACTAGGCTCAGCGGTTC	58.90	133
	R CGCGAGGAGTAGGATTGTGA	59.26	
LbPIP2;12	F CTTGATCTCCCCTGGCTACA	58.50	219
	R ACCAAGAACGCTGCAAATCC	59.40	
LbPIP1;6	F TGGTGCTGGTGTTGTCAAAG	58.90	133
	R GACAAGAACAAAGGTGCCGA	58.69	
LbPIP1;2	F AACCAGGGCAGTGTTCTACA	58.86	120
	R CTCACAACATTGGCACCTCC	59.12	
LbPIP1;5	F TCGGAGAGGGCACATTAACC	59.46	133
	R ACCTTTGACAACACCAGCAC	58.90	
LbPIP1;3	F TTGGGTACTGCTGCACAAAC	58.97	110
	R TCAGCAATTCCAGCCCTGTA	59.01	
LbPIP1;1	F CTTGGTGCTATCTGTGGTGC	58.91	120
	R AGCACCAAGTCCATCACCTT	59.23	
LbPIP1;4	F ACTGATGCCAAGAGAAACGC	58.84	106
	R CGGTAATTGGGATGGTTGCC	59.26	
LbPIP2;11	F CAGTGGCGTACATGATGGTG	58.99	139
	R AATGCTACGCCCCTTGAGTA	58.80	
LbPIP2;9	F CCAGCAGTGACATTTGGGTT	58.67	150
	R ACCACCACCACCTATGTTGT	58.85	
LbPIP2;8	F CTTCTTGGTATCGCTTGGGC	58.98	100
	R CGAATGTCACTGCTGGGTTA	57.91	
LbPIP2;6	F TACACTGATCCACCTCCTGC	58.80	191
	R AGTATACCAACACCACCGCA	59.02	
LbPIP2;4	F TCTGGAGGACACATCAACCC	59.02	134
	R AAAGCCTTGACGAAACCCAC	58.97	
LbPIP2;10	F GAGCTGCCGTTATCCTCAAC	58.71	147
	R TCCTGAATGAACCAAGGGCT	58.92	
LbPIP2;7	F TCTTGGCTCCACTTCCCATT	58.92	232
	R CCAAGAGGTTTGATGGCACC	59.11	
LbPIP2;5	F TGCTGAGTTCATTGCCACAC	59.05	447
	R GGGTCAGTGGCAGAAAAGAC	58.76	
LbPIP2;1	F ATGACTAAGGACGTTGAGGT	50.90	110
	R CTGTAAAATGACCATTTTCC	49.90	
LbPIP2;2	F ATGGGTAAAGACATCGAAGT	51.10	110
	R CTGTAAAATGACCATTTTCC	49.90	
LbACTIN1	F CTCAGCACCTTCCAGCAGAT	57.50	

R TAACACTGCAACCGCATTTC	57.10
